# *MFAP5* Activates *ITGA5* to Drive Tooth Germ Mineralization Through the MAPK/ERK Pathway: Insights from Single-Cell Transcriptomics

**DOI:** 10.3390/ijms27010394

**Published:** 2025-12-30

**Authors:** Xu Wang, Lanxin Gu, Ping Zhang, Yongsheng Zhou

**Affiliations:** 1 Department of Prosthodontics, Peking University School and Hospital of Stomatology, 22 Zhongguancun South Avenue, Haidian District, Beijing 100081, China; 2 National Center for Stomatology, National Clinical Research Center for Oral Diseases, National Engineering Research Center of Oral Biomaterials and Digital Medical Devices, 22 Zhongguancun South Avenue, Haidian District, Beijing 100081, China; 3 Beijing Key Laboratory of Digital Stomatology, National Health Commission Key Laboratory of Digital Technology of Stomatology, 22 Zhongguancun South Avenue, Haidian District, Beijing 100081, China; 4 Institute of Advanced Clinical Medicine, Peking University, No.38 Xueyuan Road, Haidian District, Beijing 100191, China

**Keywords:** MFAP5, odontogenesis, MAPK signaling

## Abstract

Tooth germ development is a precisely orchestrated process dependent on integrated cellular interactions and molecular signals, yet its regulatory mechanisms remain incompletely defined. Here, we constructed a high-resolution cellular atlas of miniature pig tooth germs using 10× single-cell RNA sequencing to investigate the molecular mechanisms underlying tooth mineralization. By leveraging cellular heterogeneity and dynamic gene expression trajectories in epithelial and mesenchymal populations, we identified microfibril-associated protein 5 (MFAP5) as a previously unrecognized regulator of the odontogenic program. Functional assays demonstrate that MFAP5, an extracellular matrix component, is indispensable for mesenchymal differentiation and matrix mineralization in vitro. Mechanistically, MFAP5 engages Integrin alpha-5 (ITGA5) to activate Extracellular Signal-Regulated Kinase/Mitogen-Activated Protein Kinase (ERK/MAPK) signaling in odontoblast-lineage cells, thereby promoting odontoblast differentiation and dentin deposition. Collectively, our single-cell–resolved analyses uncovered a MFAP5–ITGA5–ERK/MAPK signaling axis that operates in a cell-state–specific manner during tooth germ mineralization, providing new mechanistic insights into odontogenic differentiation and a potential molecular basis for dental tissue regeneration strategies.

## 1. Introduction

Tooth development is a highly dynamic and complex process, driven by the coordinated actions of multiple cell populations and gene regulatory networks. Early morphogenesis of tooth germ originates from interactions between ectodermal epithelium and condensed mesenchyme [1,2,3], progressing through the bud, cap, and bell stages, and giving rise to the enamel organ, dental papilla, and dental follicle, from which enamel, dentin, cementum, dental pulp, periodontal ligament, and alveolar bone are ultimately derived [4,5]. The majority of dental hard tissues result from the mineralization of the surrounding extracellular matrix, rendering tooth germ mineralization a critical event for the structural and functional integrity of teeth. This process is orchestrated by classical signaling pathways, including fibroblast growth factor (FGF), bone morphogenetic protein (BMP), Wingless/Integrated (Wnt), and sonic hedgehog (Shh) pathways [6,7,8,9]. Recent studies have further highlighted the role of mechanotransduction signaling pathways, such as Yes-associated protein and transcriptional coactivator with PDZ-binding motif (YAP/TAZ) [10,11], phosphoinositide 3-kinase-protein kinase B (PI3K-AKT) [12], and Extracellular Signal-Regulated Kinase/Mitogen-Activated Protein Kinase (ERK/MAPK) [13,14], in regulating tooth development. These signaling cascades and gene regulatory networks coordinate the precise cellular behaviors required for proper tooth morphogenesis and function.

The ECM serves as a dynamic scaffold that not only provides a framework for tissue mineralization but also stores biochemical factors and transmits mechanical signals to regulate cellular differentiation [15,16,17]. Microfibril-associated protein 5 (MFAP5), a component of the MAGP family, is integral to the microfibrillar niche alongside fibrillins and other matrix elements [18]. MFAP5 has been implicated in elastic fiber assembly, adipose metabolism, angiogenesis, and bone formation [19,20,21,22,23]. Both MFAP2 and MFAP5 are strongly linked to skeletal biology: *MFAP2* knockout mouse models exhibit age-related bone loss [24,25,26], while in vivo overexpression of *MFAP5* increases bone density [19]. At the cellular level, MFAP5 expression correlates with osteogenic biomarkers such as *Runx2*, *Col1a1*, and *Msx*-2 [27,28,29]. Since both bone formation and tooth development rely on ECM remodeling and mineralization, we hypothesized that *MFAP5* may also play a crucial role in tooth germ mineralization.

Recent advances in single-cell transcriptomics have enabled high-resolution dissection of cell heterogeneity and gene expression dynamics during tooth development [30,31,32]. However, existing models have limitations: mice lack permanent dentition; human tooth germ samples are difficult to obtain and constrained by ethics; and organoid models are variable in reproducibility. Miniature pigs, in contrast, closely resemble humans in tooth replacement, germ morphology, and mineralization patterns [33,34,35]. In this study, we utilized single-cell RNA sequencing to map the cellular and molecular landscape of tooth germ development across four stages in miniature pigs and identified MFAP5 as a key regulator of mineralization through the ITGA5–ERK/MAPK pathway. These findings provide new mechanistic insights into ECM-mediated regulation of odontogenic differentiation and offer a molecular framework for future therapeutic and regenerative strategies in dental medicine.

## 2. Results

### 2.1. Cellular Landscape of the Developing Tooth Germ

**Figure 1:**
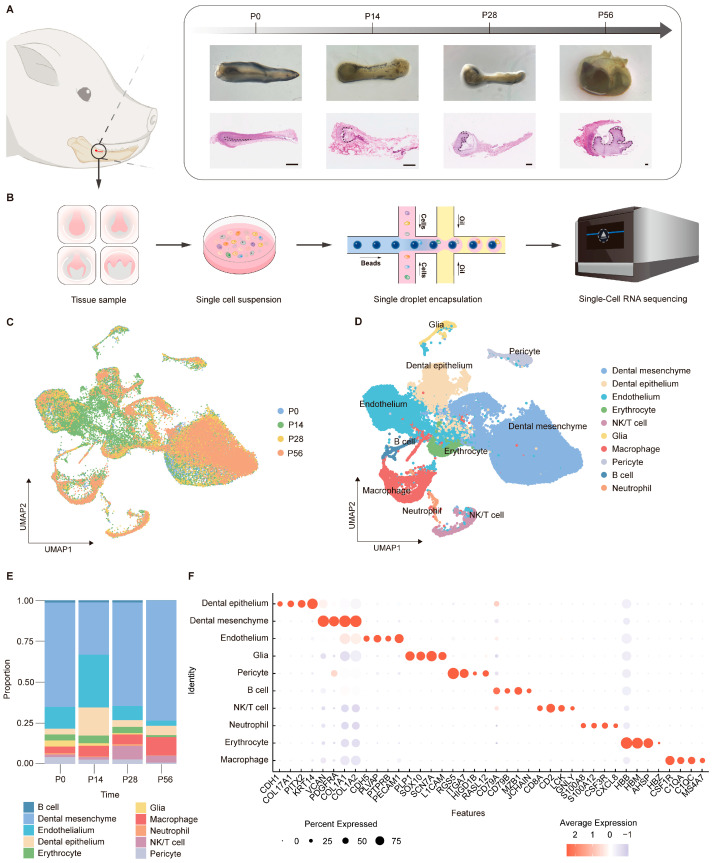
Single-cell transcriptomic map across developmental stages of tooth germ. (**A**) Schematic illustration of tissue sampling sites in the miniature pig mandible (left panel), stereomicroscope images, and corresponding H&E staining sections (right panel) showing tooth development at four key stages (scale bars: 100 μm). (**B**) Workflow diagram of single-cell RNA sequencing using tooth germ tissues (*n* = 5 for each group). (**C**) Uniform manifold approximation and projection (UMAP) visualization of integrated single-cell transcriptomic data, colored by developmental stage. (**D**) UMAP visualization of integrated single-cell transcriptomic data, colored by cell type. (**E**) Dot plots showing the expression of selected marker genes across cell types. Dot size represents the proportion of expressing cells, and color indicates average expression level. (**F**) Bar plot of cell type distribution across all samples.

To delineate the cellular composition and dynamic changes during tooth germ development, we performed single-cell RNA sequencing (scRNA-seq) on mandibular second permanent molar tooth germs from miniature pigs at four different stages: bud (postnatal day 0, P0), cap (P14), bell (P28), and secretory/mineralization stage (P56) (Figure 1A). Stereomicroscopy and hematoxylin-eosin (H&E) staining confirmed the morphological identity of each stage. After tissue dissociation, quality control, and data integration, a total of 49,937 high-quality single cells were retained for downstream analysis (Figure 1B).

Unsupervised clustering and uniform manifold approximation and projection (UMAP) analysis revealed a diverse cellular landscape comprising distinct epithelial, mesenchymal, endothelial, immune, and neural-associated populations (Figure 1C,D). Quantitative analysis of cell-type composition revealed stage-dependent shifts in cellular proportions during tooth germ development (Figure 1E). At P0, mesenchymal cells constituted the largest fraction, whereas epithelial populations increased at P14. At later stages (P28 and P56), mesenchymal cells progressively dominated the cellular landscape, consistent with advancing tissue maturation [36,37,38,39]. Dot plot analysis of canonical marker genes demonstrated highly cell-type-specific expression patterns across epithelial, mesenchymal, vascular, neural, and immune populations (Figure 1F), supporting a well-defined and dynamically regulated cellular landscape during tooth germ development. Together, these results establish a comprehensive single-cell atlas of tooth germ development in a large-animal model and provide a foundation for subsequent analyses of stage-specific molecular programs, particularly those associated with mesenchymal maturation and matrix mineralization.

### 2.2. Transcriptomic Diversity and Dynamic Changes in Dental Epithelial Cells

**Figure 2:**
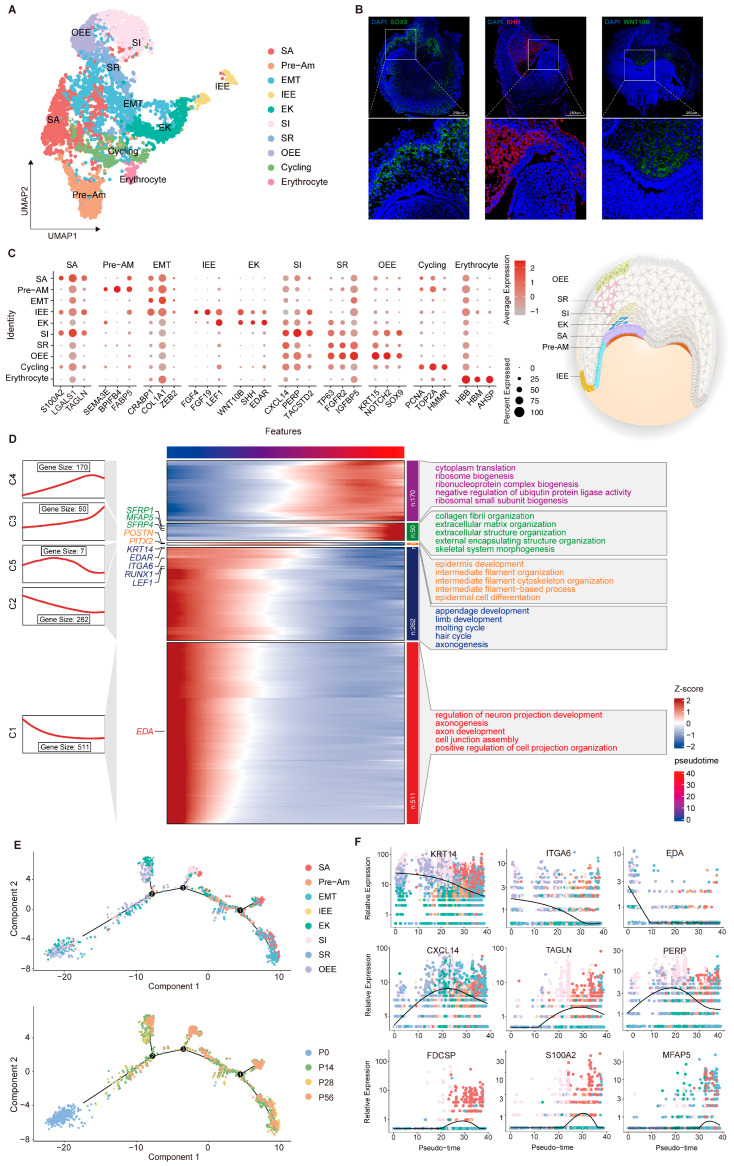
Developmental dynamics of dental epithelial subpopulations during tooth development. (**A**) UMAP of dental epithelial subpopulations with coloring based on cell type annotation. (**B**) Validation of specific marker expression in distinct subpopulations by immunofluorescence. (**C**) Dot plot visualization of representative markers across defined subpopulations. (**D**) Integrated analysis of gene expression dynamics and functional annotation in dental epithelial subpopulations. (**E**) Pseudotime trajectory constructed from epithelial cell clusters. The numbers (1, 2 and 3) shown in (**E**) represent the pseudotime branches inferred by Monocle 2, indicating distinct transcriptional trajectories along the differentiation process (**F**) Expression dynamics of selected marker genes along pseudotime, illustrating stage-specific transcriptional changes during dental epithelial differentiation.

To better understand the epithelial compartment during tooth development at higher resolution, we performed subclustering of epithelial cells and identified 11 transcriptionally distinct subpopulations (Figure 2A). Cells expressing *SHH*, *WNT*, and *EDAR* were annotated as the enamel knot [9,40,41,42], a transient epithelial signaling center that regulates cell proliferation, differentiation, and migration, thereby guiding epithelial folding and cusp formation (Figure 2C). During the bell stages, epithelial tissue undergoes stratification, intercalation, invagination, and canopy contraction, forming the enamel organ [43,44]. This primarily involves the inner (IEE) and outer (OEE) enamel epithelium and the stellate reticulum (SR) that they surround. The IEE group shows strong enrichment of *FGF4*, *FGF19*, and *LEF1*, functioning as the progenitor compartment that generates preameloblasts and initiates ameloblast differentiation [45,46]. In contrast, *IGFBP5*, *TP63*, *KRT15*, *NOTCH2*, and *SOX9* are preferentially expressed within the OEE and SR group, reflecting their identity as basal progenitor cells and epithelial support cells [37,47,48,49]. In the late bell stage, the IEE cells respond to mesenchymal cues and differentiate into the stratum intermedium (SI), pre-ameloblast (PA), secretory ameloblast (SA) and maturation ameloblast (MA) [1]. Differentiating ameloblasts exhibit stepwise transcriptional signatures, with markers such as *TACSTD2*, *TAGLN*, *FABP5*, *SEMA3E*, *BPIFB4*, *S100A6* and *S100A2* delineating successive steps of lineage commitment and functional maturation. [50,51,52,53,54]. Immunofluorescence microscopy was performed to validate the identified cell populations using antibodies against WNT10B, SHH, and SOX9 (Figure 2B). Differential expression analysis combined with Gene Ontology (GO) annotation revealed a dynamic transcriptional trajectory, with early-stage genes enriched in epithelial development pathways (e.g., epidermis development) and later stages shifting toward collagen fiber organization and extracellular matrix assembly, revealing the onset of enamel mineralization (Figure 2D).

Pseudotime analysis was performed to investigate the continuous differentiation trajectory across the predefined epithelial clusters (Figure 2E). This analysis positioned P0 progenitors at the root of the lineage trajectory. The first bifurcation was populated mainly by EK cells, followed by the subsequent branch, which consisted primarily of SI clusters. Cells remaining on the principal axis transitioned through pre-ameloblasts and ultimately matured into secretory ameloblasts. Based on the inferred trajectory, we examined the temporal expression patterns of several epithelial markers. The peak expression of *KRT14* and *ITGA6* at the outset confirms their function as progenitor factors in early tooth development [55,56]. In contrast, *MFAP5* and *S100A2* displayed a transient upregulation during the differentiation phase, a pattern consistent with the progression of enamel mineralization (Figure 2F). Collectively, these results delineate the refined organization and stage-dependent functional transitions of the dental epithelium lineage, establishing a systematic framework for further exploration of epithelial morphogenesis mechanisms.

### 2.3. Transcriptomic Diversity and Dynamic Changes in Dental Mesenchymal Cells

**Figure 3:**
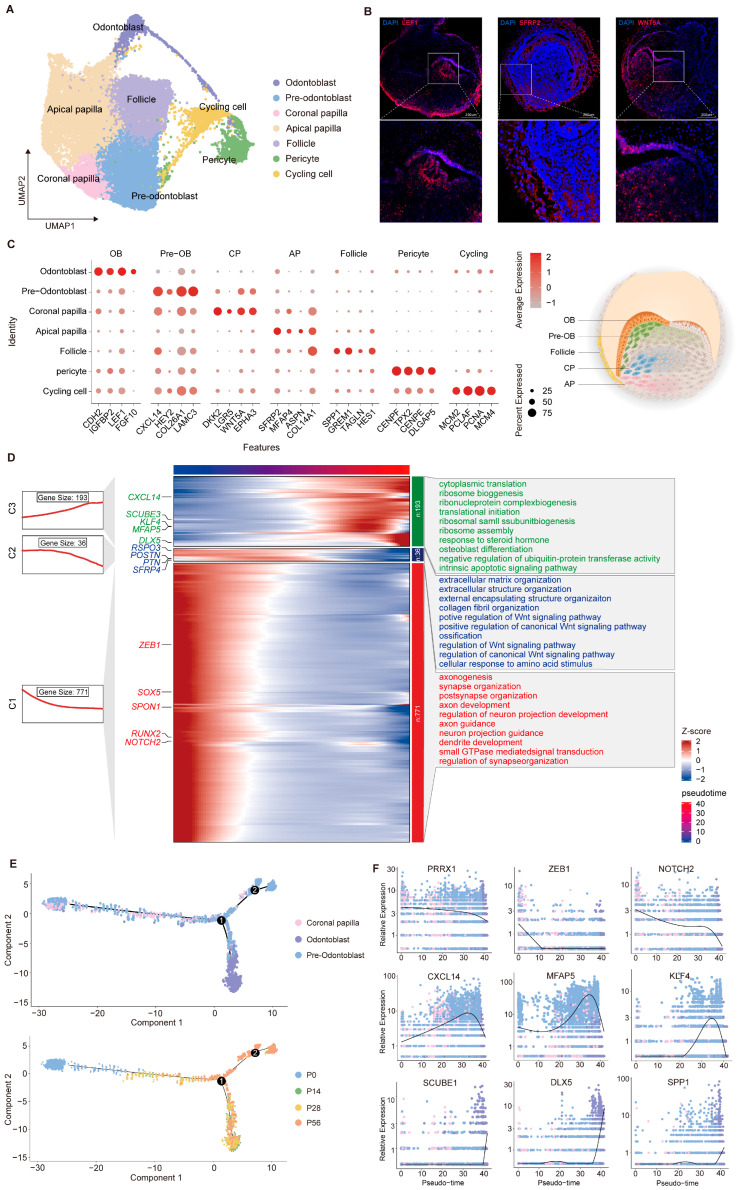
Developmental dynamics of dental mesenchymal subpopulations during tooth germ morphogenesis. (**A**) UMAP of dental mesenchymal subpopulations with coloring based on cell type annotation. (**B**) Validation of specific marker expression in distinct mesenchymal subpopulations via immunofluorescence. (**C**) Dot plot visualization of representative markers across defined subpopulations. (**D**) Integrated analysis of gene expression dynamics and functional annotation in dental mesenchymal subpopulations. (**E**) Pseudotime trajectory constructed from mesenchymal cell clusters. The numbers (1 and 2) shown in (**E**) represent the pseudotime branches inferred by Monocle 2. (**F**) Expression dynamics of selected marker genes along pseudotime, illustrating stage-specific transcriptional changes during dental mesenchymal differentiation.

The dental mesenchyme constitutes the other principal compartment of the tooth germ, serving as a source of inductive cues and reciprocal signals that specify epithelial fate and coordinate tooth morphogenesis. To resolve its developmental organization, we next characterized distinct mesenchymal subsets at single-cell resolution (Figure 3A). Upon entry into the cap stage, mesenchymal cells segregate into two principal lineages, one of which envelops the epithelial and papillary compartments and gives rise to the dental follicle. In our dataset, the dental follicle can be distinguished by the expression of marker genes such as genes *SPP1*, *GERM1*, and *HES1* and will later form supporting structures including the periodontal ligament, alveolar bone, and cementum [57,58,59]. As development progresses through the bell and secretory stages, the dental papilla subdivides into four transcriptionally distinct subpopulations (Figure 3C). Odontoblasts (*FGF10*, *LEF1*, and *CDH2*) and pre-odontoblasts (*CXCL14*, *LAMC3*, *HEY2*) represent the terminal and intermediate stages of mesenchymal differentiation, whereas coronal papilla cells (*WNT5A*, *DKK2*, *LGR5*) and apical papilla cells (*SFRP2*, *ASPN*, *MFAP4*) correspond to earlier or more distal progenitors [38,54,60,61,62]. In vivo fluorescence imaging revealed spatial expression patterns that closely matched our scRNA-seq data, with LEF1 predominantly localized to the anterior tooth papilla and SFRP2 confined to the posterior region. We then mapped the temporal dynamics of gene expression to delineate the functional programs underlying dental papilla development, identifying three transcriptional waves, each enriched for specific regulatory pathways and biological processes, including Wnt signaling, extracellular matrix organization, and osteoblast differentiation (Figure 3D).

To clarify the continuous differentiation trajectory of dental papilla, we reconstructed these populations and performed pseudotime inference using Monocle 2 [63] (Figure 3E). This analysis revealed stepwise progression from early mesenchymal progenitors to the odontoblast lineage. More precisely, stemness-associated genes (*NOTCH2* and *PRRX1*) were predominantly expressed in the early phase [64]; intermediate-stage genes (KLF4 and *MFAP5*) peaked during lineage commitment [65,66]; and genes related to mineralization, including *SPP1* and *DLX5*, were upregulated during the terminal differentiation phase, reflecting the ordered nature of odontoblast maturation [67,68,69] (Figure 3F). Together, these findings reveal a hierarchical organization of dental mesenchymal differentiation and define the transcriptional programs that drive tooth development.

### 2.4. MFAP5 in the Mineralization Maturation of Dentin Cells

**Figure 4:**
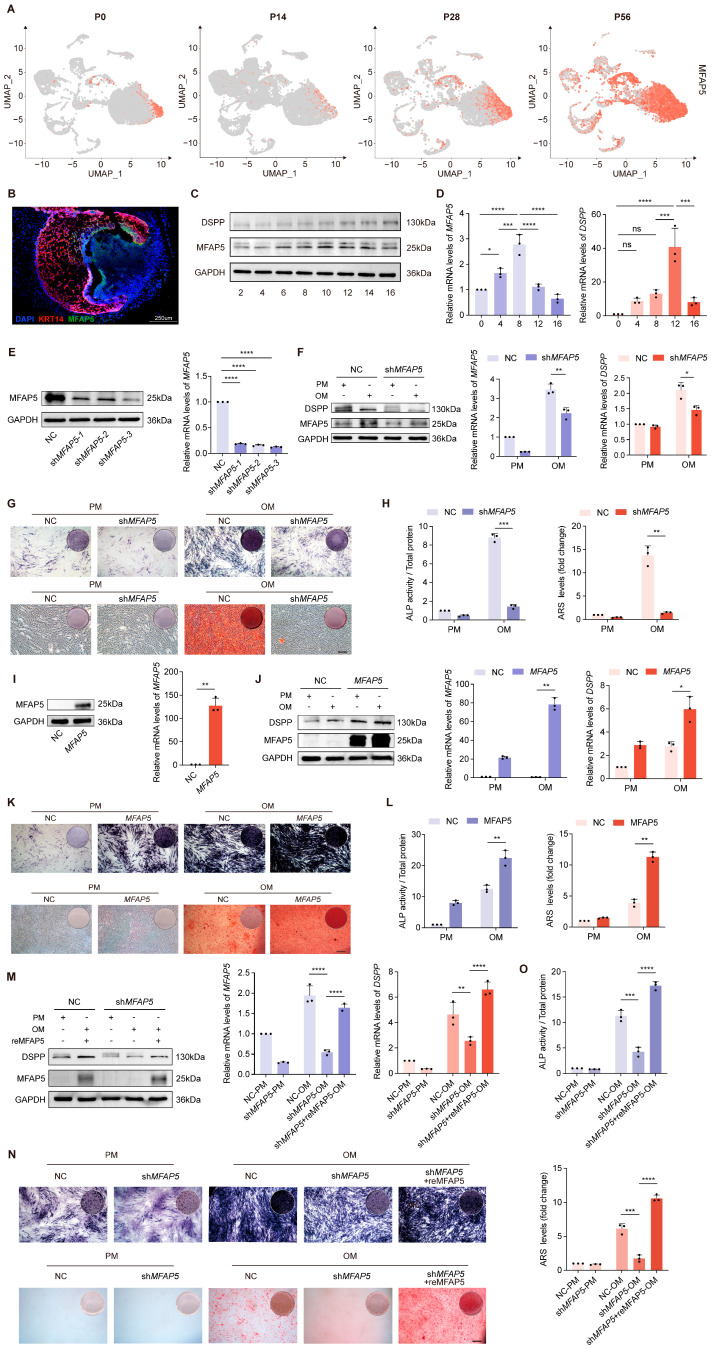
*MFAP5* deficiency impairs odontogenic differentiation of hDPSCs. (**A**) Feature plots showing MFAP5 expression at four stages. (**B**) Immunofluorescence staining of P28 molars for MFAP5 (green), KRT14 (red), and DAPI (blue), showing increased MFAP5 expression during early postnatal tooth development. Representative images from three biological replicates. (**C**) Western blot examination and protein quantification of MFAP5 expression in hDPSCs following differentiation induction. GAPDH was used for normalization. (**D**) qRT-PCR analysis showed similar expression trends in hDPSCs. GAPDH was used for normalization. (**E**) Assessment of *MFAP5* knockdown efficiency by qRT-PCR and Western blot. (**F**) MFAP5 and DSPP protein expressions were determined by qRT-PCR and Western blot after 7 days of odontogenic induction. GAPDH was used for normalization. (**G**) Staining and quantification of ALP in NC and sh*MFAP5* hDPSCs. (scale bars: 100 μm) (**H**) ARS staining and quantification analysis of NC and sh*MFAP5* hDPSCs. Regulates Odontogenic Differentiation of hDPSCs. (**I**) Assessment of *MFAP5* overexpression efficiency by qRT-PCR and Western blot. (**J**) Odontogenic differentiation potential of control and *MFAP5* hDPSCs was evaluated by qRT-PCR and Western blot. GAPDH was used for normalization. (**K**) ALP and ARS staining of control and MFAP5 hDPSCs. (scale bars: 100 μm) (**L**) Quantification analysis of ALP and ARS staining. (**M**) sh*MFAP5* hDPSCs were rescued by transfection with recombinant MFAP5. (**N**) MFAP5 rescue restored osteogenic potential of knockdown hDPSCs, indicated by ALP and ARS staining. (scale bars: 100 μm) (**O**) ALP and ARS results indicating enhanced mineral deposition following MFAP5 rescue. Quantitative data are presented as mean ± SD (*n* = 3). Statistical significance is indicated as: * *p* < 0.1, ** *p* < 0.01, *** *p* < 0.001, **** *p* < 0.0001; ns: not significant.

Single-cell transcriptomic profiling revealed active involvement of *MFAP5* in both epithelial and mesenchymal differentiation within the tooth germ, a distribution pattern also noted in previous studies [54]. In our dataset, *MFAP5* was upregulated in the mesenchyme during odontoblast commitment, suggesting a potential role in their differentiation. To verify this hypothesis, *MFAP5* expression across four developmental stages was examined using UMAP visualization (Figure 4A). Immunofluorescence staining further validated this trend, showing prominent localization of MFAP5 at the epithelial–mesenchymal junction at the P28 stage (Figure 4B). Notably, pseudotime analysis indicated that MFAP5 expression decreased during later stages of dentin matrix secretion and mineralization (Figure 3F), consistent with protein expression patterns observed in human dental pulp stem cells (hDPSCs), a mesenchymal cell model of tooth germs (Figure 4C). Quantitative PCR further corroborated this dynamic pattern, showing an initial increase followed by a decrease in MFAP5 expression during induced differentiation (Figure 4D).

To explore the impact of *MFAP5* in odontoblast differentiation and mineralization, we generated *MFAP5* stable knockdown cells and confirmed transduction efficiency (Figure 4E). One week after mineralization induction, Western blot and qRT-PCR analysis indicated a significant reduction in the expression of the mineralization marker DSPP in *MFAP5*-deficient cells (Figure 4F). Correspondingly, ALP activity and mineralized matrix formation were markedly inhibited (Figure 4G,H). Conversely, overexpression of MFAP5 in hDPSCs via viral transfection significantly increased both protein and mRNA levels of MFAP5 (Figure 4I). Following differentiation induction, cells overexpressing MFAP5 exhibited higher DSPP expression compared to control cells (Figure 4J), and staining experiments demonstrated enhanced ALP activity and mineralized nodule formation (Figure 4K,L). Furthermore, supplementation of recombinant MFAP5 protein to *MFAP5*-deficient cells substantially restored DSPP expression and promoted mineralized nodule formation (Figure 4M–O). These results indicate that *MFAP5* plays a key regulatory role in odontoblast mineralization, with its expression directly modulating the activation of mineralization markers and the formation of mineralized nodules.

### 2.5. MFAP5 Regulates MAPK Signaling via ITGA5 Dependency

**Figure 5:**
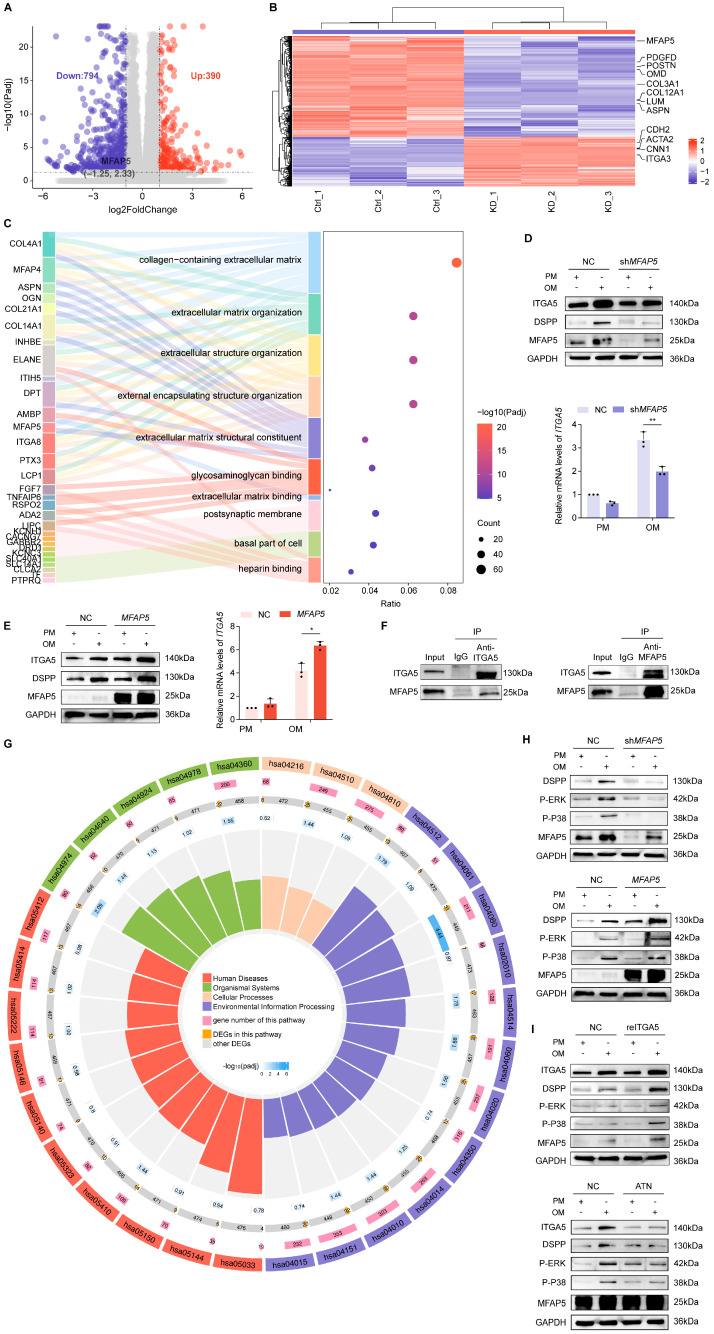
*MFAP5* promotes ERK signaling by regulating *ITGA5* expression. (**A**) Volcano plot showing the comparison of differentially expressed genes between control and sh*MFAP5* hDPSCs, with *MFAP5* highlighted. (**B**) Heatmap of differentially expressed genes between control and knockout groups. (**C**) Sankey diagram of the top 10 enriched GO biological processes (BP) among DEGs. (**D**) *MFAP5* knockdown decreases ITGA5 protein abundance, revealed by Western blot. (**E**) *MFAP5* overexpression increases ITGA5 protein levels as shown by Western blot. (**F**) Co-IP assay indicating interaction between MFAP5 and ITGA5 proteins. (**G**) Circle chart of KEGG pathway enrichment analysis of DEGs. (**H**) Western blot showing that *MFAP5* knockdown and overexpression modulate ERK phosphorylation. (**I**) Rescue of ERK phosphorylation by ITGA5 overexpression in sh*MFAP5* hDPSCs demonstrated by Western blot. Quantitative data are presented as mean ± SD (*n* = 3). Statistical significance is indicated as: * *p* < 0.1, ** *p* < 0.01.

To characterize the molecular mechanisms underlying MFAP5-mediated regulation of odontogenic differentiation and mineralization, we performed RNA sequencing (RNA-seq) on *MFAP5*-deficient and control hDPSCs. A total of 808 differentially expressed genes (DEGs) were identified following *MFAP5* knockdown, as depicted in the volcano plot (Figure 5A). Several genes involved in ECM organization and odontogenic mineralization, including *COL1A1*, *SPP1*, and *MMP2*, were highlighted in the heatmap (Figure 5B). Gene Ontology (GO) enrichment indicated *MFAP5* deficiency predominantly affected ECM-related pathways, suggesting that *MFAP5* may regulate odontoblast differentiation via ECM-associated signaling cascades (Figure 5C). Since ECM primarily modulates cellular programs via integrins, which detect and relay chemical and mechanical cues, we next examined whether the effects of *MFAP5* are mediated by integrin signaling [17,70]. Given that MFAP5 contains an Arginine-Glycine-Aspartic acid (RGD) motif, we identified ITGA5 as a candidate receptor among the RGD-binding integrins [71,72]. Upon odontoblastic induction, *ITGA5* expression was markedly reduced in *MFAP5* knockdown cell lines at both the transcript and protein levels, whereas *MFAP5* overexpression led to a corresponding increase in *ITGA5* expression, as determined by quantitative RT–PCR and Western blot analyses (Figure 5D,E). These results confirm that ITGA5 is positively regulated by MFAP5. Co-immunoprecipitation (Co-IP) experiments further revealed a physical association between MFAP5 and ITGA5 (Figure 5F), providing a structural basis for coordinated signal transduction.

Kyoto Encyclopedia of Genes and Genomes (KEGG) pathway analysis revealed that *MFAP5* dysregulation impacts multiple pathways implicated in mineralization, including MAPK, WNT, HIPPO, and PI3K, demonstrating its role in coordinating tooth morphogenesis and organogenesis (Figure 5G). Consistently, *MFAP5* knockdown attenuated phosphorylation of p38 and ERK at the protein level, whereas *MFAP5* overexpression enhanced their activation under mineralization induction, supporting an essential role of MFAP5 in promoting odontogenic differentiation through the ERK/MAPK pathway (Figure 5H). To dissect the functional relevance of *ITGA5* in MAPK activation, we employed both pharmacological inhibition and recombinant protein supplementation experiments. In *MFAP5* knockdown cells, recombinant ITGA5 restored ERK and p38 activation, concomitantly rescuing DSPP expression. Conversely, treatment of *MFAP5*-overexpressing hDPSCs with ATN markedly suppressed ERK and p38 phosphorylation and inhibited DSPP expression (Figure 5I). Taken together, these findings demonstrate that MFAP5 promotes odontogenic differentiation and mineralization by activating the ERK/MAPK signaling pathway in an ITGA5-dependent manner, highlighting its critical role in ECM-mediated signal transduction during tooth development.

## 3. Discussion

In mammalian craniofacial development, tooth germs are formed through a series of coordinated signaling events driven by reciprocal interactions between the invaginated oral epithelium and the underlying mesenchyme [73]. Although extensive research has identified key signaling molecules and specific stem cell types at different stages of tooth germ development, the roles of certain molecular functions and cell types remain poorly understood throughout the full developmental process. Traditional approaches, such as histology, immunofluorescence, and gene knockout models, have inherent limitations in fully capturing the cellular heterogeneity and dynamic changes that occur during tooth development. With the advent of single-cell sequencing technologies [74,75], it has become possible to systematically define cellular composition and delineate specific gene expression programs.

Numerous single-cell transcriptomic studies in mouse teeth have provided detailed catalogs of epithelial, mesenchymal, and neural crest-derived populations [76,77], but they primarily focus on static cell annotation [78] and progenitor characterization [79]. Fundamental differences in tooth type and developmental patterning between mice and humans also limit the direct applicability of these findings to human tooth development. Human tooth germ single-cell studies remain scarce due to limited sample availability and ethical constraints, with most datasets derived from adult third molars [80,81] or from periodontal ligament tissues [82,83], providing only partial snapshots of dental development. To date, only a few studies have performed single-cell analyses across multiple sequential stages of human tooth development [84,85]. In the present study, our single-cell data from four key developmental stages of miniature pig permanent molars enabled the construction of a continuous atlas, providing a comprehensive framework that closely recapitulates human tooth development. More importantly, we leverage this atlas as a functional basis to precisely identify key regulators of differentiation, enabling the transition from descriptive resource to mechanistic insight.

Our data covers four major developmental stages: bud (P0), cap (P14), bell (P28), and secretory mineralization (P56), and presents the cellular specificity and molecular diversity across the entire tooth germ development cycle, with a focus on the two main cell types—epithelial and mesenchymal cells. Specifically, we first characterized distinct functional clusters and their molecular signatures across each developmental stage and further revealed the dynamic changes and mechanistic pathways of key genes associated with tooth formation. The enamel knot, a signaling center representing tooth development, coordinates the formation of the tooth cusp by sending signals to adjacent cells. In line with known classic markers, this is characterized by the high expression of *SHH*, *EDAR*, and *WNT10B* [86,87]. Cells surrounding the enamel knot, including IEE and SI, which serve as a primary source for the evolution of epithelial cells towards ameloblasts, express molecules such as *FGF4*, *FGF19*, and *TACSTD2* [45,46]. Ameloblasts, which are directly involved in enamel formation, produce proteins associated with matrix secretion and mineralization. Similarly, we conducted the lineage classification and evolutionary trajectory analysis in dental papilla. The cells at various differentiation stages displayed distinct functional features, with molecular differences closely tied to their specific roles in tooth development. In the early stages, genes associated with proliferation and early differentiation, such as *PRRX1*, *NOTCH2*, and *ZEB1*, were prominently expressed. As development progressed, gene expression shifted toward factors related to mineralization, including *KLF4*, *MFAP5*, and *DLX5*, revealing key molecular decisions in dentin formation [65,66]. These molecular differences provide insights into tooth structure development, linking gene expression patterns to cellular roles in odontogenesis.

Our results indicate that the crown development process is accompanied by stage-specific gene programs, among which MFAP5 exhibits prominent expression during the mineralization and extracellular matrix deposition phases, suggesting a potential role in tooth formation. MFAP5 is a member of the microfibril-associated glycoprotein family, originally identified and localized by Gibson et al. in the nuchal ligament of fetal bovine cervical tissue, and is known to contribute to the structural organization of the extracellular matrix (ECM) [88,89]. In our sequencing data, *MFAP5* expression mirrored that of mineralization markers [54], showing elevated levels in both dental epithelial and mesenchymal cells at the cap stage compared to the bud stage. These temporal and spatial expression patterns position MFAP5 not only as a structural scaffold within the ECM but also as a signaling molecule that coordinates cell fate decisions and morphogenetic processes. Through in vitro experiments using hDPSCs, we verified that *MFAP5* expression is closely associated with the early differentiation of odontoblasts during tooth development. The creation of *MFAP5* knockdown and overexpression cell lines further confirmed the pivotal role of *MFAP5* in regulating mineralization in hDPSCs.

Mechanistic investigations via RNA-seq revealed that *MFAP5* modulation regulates ECM-related signaling pathways during hDPSC differentiation. Given that integrins are central receptors for ECM signaling and the presence of an RGD motif within MFAP5, we hypothesized that MFAP5 might act on a specific type of integrin. Previous studies have also demonstrated that MFAP5 can interact with integrins via its RGD domain, thereby modulating cellular perception of the ECM [71,90]. Subsequent functional experiments confirmed that MFAP5 directly interacts with ITGA5, and the modulation of MFAP5 levels was accompanied by corresponding changes in ITGA5 protein expression. KEGG analysis revealed that *MFAP5* modulation affects multiple pathways, including MAPK, WNT, HIPPO, and PI3K signaling, highlighting the complexity of tooth development [91,92]. A similar paradigm has been described for members of the Small integrin-binding ligand N-linked glycoprotein (SIBLING) family, such as DMP1, DSPP, and osteopontin, which function as extracellular matrix-associated ligands that engage integrin-mediated signaling to regulate mineralization [93,94]. Rather than operating through a single linear pathway, SIBLING proteins are known to activate multiple downstream cascades, including ERK/MAPK, c-Jun N-terminal kinase (JNK) and PI3K-Akt [95,96,97]. To validate the RNA-seq findings, we performed functional experiments in hDPSCs and found that changes in MFAP5 expression were consistently associated with MAPK pathway activity during mineralization. ERK signaling was prioritized based on its consistent activation downstream of MFAP5 and ITGA5 modulation and its clear functional association with odontogenic outcomes [98,99,100]. In contrast, although p38 activation was also observed, its specific functional contribution to odontoblast mineralization was not systematically dissected in the current study [101]. Furthermore, our experiments also substantiated that MFAP5 modulates the ERK/MAPK pathway via ITGA5, thereby modulating the mineralization capacity of hDPSCs.

In conclusion, by dissecting transcriptional regulation at single-cell resolution, we have delineated the role of the MFAP5–ITGA5–ERK/MAPK signaling axis in odontogenic differentiation. These mechanistic insights may guide novel therapeutic targets for dental regeneration and help establish a mineralization-permissive microenvironment for stem cell–based regenerative engineering.

## 4. Materials and Methods

### 4.1. Experimental Animal Ethics and Sample Collection

All experiments involving miniature pigs were approved and authorized by the Institutional Animal Care and Use Committee of Peking University Health Science Center (DLASBE0242). Miniature pigs were obtained from the Institute of Zoology, Chinese Academy of Sciences (Beijing, China), approved by the Beijing Municipal Science and Technology Commission, and hold an Animal Use License (SYXK [Jing] 2017-0002) and an Animal Production License (SCXK [Jing] 2018-0005). Following euthanasia, the mandibles were carefully dissected and immediately immersed in sterile phosphate-buffered saline (PBS, Beyotime, Shanghai, China) supplemented with 8% fetal bovine serum (FBS; Gibco, Grand Island, CA, USA) and 2% penicillin-streptomycin (Gibco) at 4 °C. Under a stereomicroscope, the surrounding bone and soft tissues were meticulously removed to isolate the second molar tooth germs. Samples were collected at four distinct postnatal developmental stages (P0, P14, P28, and P56), with five biological replicates per stage.

### 4.2. Tissue Dissociation and Preparation of Single-Cell Suspensions

To isolate single cells from microdissected tooth germ tissues, all procedures were performed under sterile conditions. Each sample was gently minced into small fragments and enzymatically dissociated using a mixed solution containing neutral protease type II (4 mg/mL; Worthington Biochemical Corporation, Lakewood, NJ, USA) and collagenase type I (3 mg/mL; Worthington Biochemical Corporation) [102,103,104], followed by incubation at 37 °C for 25–30 min with gentle agitation (200 rpm) to facilitate cell release from the tissue matrix. The resulting cell suspension was passed through a 70 μm cell strainer, and the digestion was terminated by adding α-Modified Eagle’s Medium (α-MEM; Gibco, Grand Island, CA, USA) supplemented with heat-inactivated 5% fetal bovine serum (FBS; Gibco). Cells were immediately centrifuged at 1000× *g* for 5 min to collect the cells. The pellet was briefly rinsed with ice-cold Milli-Q water for less than 5 s to lyse the residual erythrocytes and immediately quenched with resuspension buffer [105]. After centrifugation, the cells were resuspended in buffer for subsequent single-cell RNA sequencing.

### 4.3. 10× Genomics cDNA Library Preparation

Cell number, viability, and doublet rate were first assessed to ensure each sample’s quality. A single-cell suspension was adjusted to a final concentration of 300 viable cells/μL and loaded into the Chromium Single Cell Controller (10× Genomics) using the Chromium Single Cell 3′ Library and Gel Bead Kit v3.1 and the Chromium Single Cell G Chip Kit (10× Genomics) to generate single-cell gel bead-in-emulsions (GEMs).

Within each GEM, mRNAs were reverse-transcribed into barcoded cDNAs. After GEM breakage, the cDNAs were recovered and amplified by PCR following the manufacturer’s protocol. The resulting sequencing libraries were assessed using an Agilent 2100 Bioanalyzer (Agilent Technologies, Santa Clara, CA, USA). The final libraries were sequenced on an Illumina NovaSeq 6000 platform (Illumina, San Diego, CA, USA) with paired-end 150 bp reads, aiming for an average depth of ~100,000 reads per cell (CapitalBio Technology, Beijing, China).

### 4.4. Single-Cell RNA Sequencing Analysis

Raw FASTQ files were processed using Cell Ranger (v3.1.0, 10× Genomics) to generate gene expression matrices. Cells expressing fewer than 200 genes or with >20% mitochondrial transcripts were excluded. After normalization and log transformation, batch effects across samples were corrected using the Harmony algorithm implemented in Seurat (v4.3.0; https://github.com/satijalab/seurat, accessed on 16 March 2025), followed by data scaling for downstream analyses. In total, 11,484, 19,250, 8508, and 10,695 high-quality single-cell transcriptomes were retained from postnatal days 0, 14, 28, and 56, respectively. Principal component analysis (PCA) was performed using the RunPCA function to capture the major sources of transcriptional variation. The top principal components were then used for neighborhood graph construction and unsupervised clustering with the FindNeighbors and FindClusters functions in Seurat. For visualization of cellular heterogeneity, dimensionality reduction was further performed using UMAP. Cluster-defining genes were identified with the FindAllMarkers function. Cell types were subsequently annotated by comparing these clusters to well-characterized cell-type markers reported in previous studies. To infer developmental progression, Monocle 2 was used to reconstruct trajectories, arranging cells along a pseudo-time axis based on gene expression dynamics and thereby revealing lineage relationships and temporal changes in cell states.

### 4.5. Lentiviral Transduction

Human dental pulp stem cells (hDPSCs) were transduced with lentiviral vectors to generate MFAP5 knockdown and MFAP5-overexpressing cell lines, together with corresponding negative control (NC) groups (Sangon Biotech, Shanghai, China). Transduction efficiency was first monitored by fluorescence microscopy, and stable cell lines were further validated by quantitative PCR and Western blot analysis. The shRNA sequences used for gene silencing are provided in Table 1.

### 4.6. Protein Extraction and Western Blotting

Total cellular proteins were extracted using RIPA lysis buffer (Santa Cruz Biotechnology, Santa Cruz, CA, USA) supplemented with protease and phosphatase inhibitor cocktails (Beijing Huaxing Boca Genetic Technology Co., Beijing, China). Protein concentrations were determined using a BCA Protein Assay Kit (Thermo Fisher Scientific, Waltham, MA, USA). Equal amounts of protein (20 μg per sample) were separated by 12.5% SDS–PAGE and transferred onto 0.22 μm PVDF membranes (Millipore, Billerica, MA, USA). The membranes were blocked for 1 h at room temperature with 10% (*w*/*v*) nonfat dry milk in Tris-buffered saline containing 0.1% Tween-20 (TBST) to prevent nonspecific binding. After washing with TBST, the membranes were incubated overnight at 4 °C with the primary antibodies: GAPDH (1:5000, Abcam, Cambridge, UK), ERK1 (phospho T202)/ERK2 (phospho T185) (1:1000, Abcam, UK), p38 (phospho T180 + Y182) (1:1000, Abcam, UK), DSPP (1:1000, Santa Cruz Biotechnology, USA), Integrin α5 (1:1000, Abcam, UK), and MFAP5 (1:1000, Abcam, UK). After three washes with TBST, membranes were incubated with species-related HRP-conjugated secondary antibodies (1:10,000, Abcam, UK) for 1 h at room temperature. Protein bands were visualized using the Omni-ECL Femto Light Chemiluminescence Kit (EpiZyme, Shanghai, China; cat. no. SQ201) and captured with a digital imaging system. GAPDH served as a loading control. Band intensities were quantified using ImageJ software (version 2.0.0-rc-69/1.52p, NIH, Bethesda, MD, USA).

### 4.7. RNA Extraction and Quantitative Real-Time Reverse Transcription PCR (qRT-PCR)

Total cellular RNA was extracted from the cells using TRIzol reagent (Invitrogen, Waltham, MA, USA), and RNA concentration and purity were assessed by Nanodrop spectrophotometry (Thermo Fisher Scientific, Wilmington, DE, USA). Only RNA samples with an A260/A280 ratio between 1.8 and 2.0 were selected for complementary DNA (cDNA) synthesis using the PrimeScript™ RT Reagent Kit (Takara, Kusatsu, Shiga, Japan). Quantitative RT-PCR analysis was conducted utilizing a 7500 RT-PCR Detection System (Applied Biosystems, Foster City, CA, USA) with SYBR Green Master Mix (YEASEN, Shanghai, China). The PCR cycling conditions were as follows: an initial denaturation at 95 °C for 10 min, followed by 40 cycles of denaturation at 95 °C for 15 s and annealing at 60 °C for 1 min. Primers were either designed using Primer-BLAST (NCBI; https://www.ncbi.nlm.nih.gov/tools/primer-blast/, accessed on 20 May 2025) or adopted from previously published studies. *GAPDH* served as the internal control, and relative expression levels were calculated using the 2^−ΔΔCt^ method. To ensure data reliability, each sample was tested in triplicate. The primers used for qRT–PCR analysis are summarized in Table 2.

### 4.8. Immunofluorescence

The extracted tissues were fixed in 4% paraformaldehyde for 24 h at 4 °C, decalcified in 10% EDTA (pH 7.4) for one week at 4 °C, and cryoprotected sequentially in 20% and 30% sucrose solutions overnight. Samples were then embedded in OCT compound (Sakura Finetek) and sectioned at a thickness of 10 μm using a cryostat. Sections were air-dried and permeabilized with 0.2% Triton X-100 (Merck KGaA, Darmstadt, Germany) for 5 min, followed by blocking with 1% bovine serum albumin (BSA; Merck KGaA, Darmstadt, Germany) for 1 h at room temperature. After three washes with PBST, sections were incubated with primary antibodies (Anti-LEF1 (Cat#ab53293, Abcam), Anti-WNT10B (Cat#ab70816, Abcam), Anti-SHH (Cat#ab53281, Abcam), Anti-WNT5A (Cat#55184-1-AP, Proteintech, Wuhan, China), Anti-SFRP2 (Cat#66328-1-Ig, Proteintech), and Anti-SOX9 (Cat#67439-1-Ig, Proteintech)) overnight at 4 °C. The next day, sections were washed and incubated with fluorophore-conjugated secondary antibodies for 2 h at room temperature in the dark. Finally, nuclei were counterstained with DAPI-containing mounting medium (Cat#ab104139, Abcam), and images were acquired using a Leica SP8 confocal microscope (Leica Corp., Wetzlar, Germany).

### 4.9. Alkaline Phosphatase (ALP) and Alizarin Red S (ARS) Staining and Quantification

HDPSCs were seeded in 12-well plates, and after being cultured or induced under the indicated conditions, cells were fixed with 95% ice-cold ethanol for 20 min at room temperature and washed three times with PBS. ALP staining was then performed according to the manufacturer’s protocol (Nanjing Jiancheng Bioengineering Institute, Nanjing, China). The colorimetric reaction was terminated at the appropriate time, and the stained cells were imaged using a scanner and a light microscope. For quantitative analysis, ALP activity was determined using a BCIP/NBT Alkaline Phosphatase Color Development Kit (CW0051, Kangwei Century, Beijing, China). The reaction product was solubilized, and absorbance was measured at 520 nm using a microplate reader. The ALP activity was normalized to the total protein concentration in each sample.

After 21 days of osteogenic induction, HDPSCs were washed gently with Milli-Q water and fixed in 95% ice-cold ethanol for 20 min [107,108,109]. Cells were stained with 1% (*w*/*v*) Alizarin Red S solution (Sigma-Aldrich, St. Louis, MO, USA, A5533), ensuring the dye completely covered the cell monolayer. After incubation for 20 min at room temperature, excess dye was removed by washing thoroughly with water. The plates were air-dried, and mineralized nodules were visualized and imaged using a scanner and a light microscope. For quantification, the bound dye was extracted by adding an equal volume of 1% (*w*/*v*) cetylpyridinium chloride (CPC, Sigma-Aldrich, C9002) solution to each well until the stain was completely dissolved. The eluted solution was transferred to a 96-well plate (100 μL per well), and absorbance was measured at 490 nm using a microplate reader (Model 7003, All-Wavelength Microplate Reader, San Jose, CA, USA). Quantitative values were calculated based on the optical density and expressed relative to control samples. Mineralization assays were conducted with three independent biological replicates (*n* = 3) per group.

### 4.10. RNA Sequencing and Analysis

RNA sequencing was performed on control (empty vector) and *MFAP5* knockdown (sh*MFAP5*) HDPSCs, and the quality was assessed by the Agilent 2100 Bioanalyzer. Sequencing libraries were subsequently prepared, evaluated for fragment size, and sequenced on an Illumina platform. Gene counts were quantified with featureCounts, and differential expression analysis was performed using DESeq2. Genes with adjusted *p* < 0.05 and fold change > 1 were considered significantly different. Functional enrichment analyses, including GO and KEGG pathways, were conducted using clusterProfiler v4.0.0.

### 4.11. Co-Immunoprecipitation (Co-IP)

Cell protein extracts were extracted using Co-IP Lysis Buffer (Thermo Fisher Scientific, Waltham, MA, USA), and the lysates were pre-cleared with magnetic beads at 4 °C for 1 h to minimize non-specific proteins. After three washes, lysates were incubated with target-specific antibodies by gentle rotation at 4 °C overnight. Protein A/G magnetic beads (MCE) were added and incubated with the immune complexes for 4 h at 4 °C. The beads were then washed three times with Co-IP buffer for 5 min each, resuspended in 2× loading buffer, and eluted by heating at 100 °C for 5 min. The precipitated complexes were resolved by SDS-PAGE and proceeded with Western blot analysis.

### 4.12. Statistical Analysis

All statistical analyses were performed using GraphPad Prism software (version 9; GraphPad Software, Inc., La Jolla, CA, USA). Data are presented as the mean ± standard deviation (SD). Comparisons between two groups were conducted using an unpaired two-tailed Student’s *t*-test, while comparisons among multiple groups were performed using one-way analysis of variance (ANOVA) followed by Tukey’s post hoc test. A *p*-value < 0.05 was considered statistically significant.

## Figures and Tables

**Table 1:** Short hairpin RNA (shRNA) sequence.

shRNA	Target Sequence
sh*MFAP5-1*	GCATCGGCCGGTTAAACAATG
sh*MFAP5-2*	GCTGCTGTTTCTTGCTGCATT
sh*MFAP5-3*	GGAGATCTGCTCTCGTCTTGT

**Table 2:** List of primers used in this study.

Gene	Forward Primer	Reverse Primer
*GAPDH*	AAGGTCGGAGTCAACGGATTTG	TCCTGGAAGATGGTGATGGGAT
*MFAP5* [106]	GGGTCAATAGTCAACGAGGAGA	CTGTAGCGGGATCATTCACCA
*DSPP*	ATATTGAGGGCTGGAATGGGGA	TTTGTGGCTCCAGCATTGTCA
*ITGA5*	GGCTTCAACTTAGACGCGGAG	TGGCTGGTATTAGCCTTGGGT

## Data Availability

The study data are available upon request from the corresponding authors.
